# Workplace Health Promotion and Wellbeing

**DOI:** 10.1155/2015/606875

**Published:** 2015-08-26

**Authors:** Lars L. Andersen, Karin I. Proper, Laura Punnett, Richard Wynne, Roger Persson, Noortje Wiezer

**Affiliations:** ^1^National Research Centre for the Working Environment, 2100 Copenhagen, Denmark; ^2^Physical Activity and Human Performance group, SMI, Department of Health Science and Technology, Aalborg University, 9220 Aalborg, Denmark; ^3^National Institute for Public Health and the Environment, P.O. Box 1, 3720 BA Bilthoven, Netherlands; ^4^VU University Medical Center, 1081 BT Amsterdam, Netherlands; ^5^University of Massachusetts Lowell, Lowell, MA 01854, USA; ^6^Work Research Centre, Dublin, Ireland; ^7^Department of Psychology, Lund University, Box 213, 221 00 Lund, Sweden; ^8^Netherlands Organisation for Applied Scientific Research (TNO), Delft, Netherlands

For most humans work is an important fact of life and something that is necessary for survival and individual wellbeing. However, the circumstances under which we work may vary considerably and are, in part, contingent on geographical location, governmental regulations, design of social welfare systems, production systems, and human resource management strategies. In many industrialized countries, demographic developments entailing an ageing workforce increase the importance of developing sustainable employments.

A healthy, safe, and productive working life is the essence of the European Agency for Safety and Health at Work's goal for employees in a modern and sustainable workplace. The goal may be obtained by ensuring the employees' wellbeing at workplaces through improvement of the working environment and through different types of health promotion initiatives at the workplace. The World Health Organization (WHO) emphasizes the workplace as a priority setting for promotion of health and wellbeing, including provision of a safe and healthy physical and psychosocial work environment. In the United States, the Total Worker Health program of NIOSH has a similar mission and emphasizes that health promotion requires compliance with health and safety regulations and protection of workers' rights as a foundation.

Workplace health promotion is the combined efforts of employers, employees, and society to improve the health and wellbeing of workers. This entails programs not only to encourage individual behavior change, but also to reduce stressors in the workplace that have “take-home” negative effects on health behaviors. However, many barriers exist for successfully developing, implementing, and evaluating health promotion and wellbeing initiatives at the workplace. For instance, small enterprises may not have the same infrastructure to support health promotion as larger companies. Some companies may lack time or knowledge to initiate and sustain meaningful health promotion initiatives. Thus, research is needed on how to develop, implement, and evaluate health promotion and wellbeing initiatives in different settings and for different groups of workers.

In this special issue, a wide array of topics on workplace health promotion and employees' wellbeing is described. The topics include (1) alternate ways to consider the value of workplace health promotion, (2) the local context needed for developing healthy workplaces, (3) the impact of participatory interventions on wellbeing at work, (4) the importance of reducing workplace stressors alongside introducing workplace health promotion, (5) strategies that supervisors use to prevent sickness absence among employees with musculoskeletal disorders, (6) motivation and barriers for workplace health promotion with physical exercise, and (7) the impact of organizational health climate on workers wellbeing.

The contributing authors' efforts have helped to make this special issue appeal to a diverse audience of researchers and illustrate well the diverse and multifaceted challenges that need to be tackled in order to make a difference. We are delighted to see the outcome of the special issue and hope that it will inspire and stimulate further research in this area.

